# RPA Combined With CRISPR/Cas12a for Rapid and Ultrasensitive Detection Dual‐Gene of Methicillin‐Resistant *Staphylococcus aureus* (MRSA)

**DOI:** 10.1002/jmr.70035

**Published:** 2026-04-19

**Authors:** Lei Chen, Jun Luo, Helin Zhang, Pingsen Zhao

**Affiliations:** ^1^ Department of Laboratory Medicine Yuebei People's Hospital Affiliated to Shantou University Medical College Shaoguan China; ^2^ Laboratory for Diagnosis of Clinical Microbiology and Infection, Yuebei People's Hospital Affiliated to Shantou University Medical College Shaoguan China; ^3^ Shaoguan Municipal Quality Control Center for Laboratory Medicine, Yuebei People's Hospital Affiliated to Shantou University Medical College Shaoguan China; ^4^ Shaoguan Municipal Quality Control Center for Surveillance of Bacterial Resistance Shaoguan China; ^5^ Shaoguan Engineering Research Center for Research and Development of Molecular and Cellular Technology in Rapid Diagnosis of Infectious Diseases and Cancer Shaoguan China

**Keywords:** CRISPR/Cas12a, detection, dual‐gene, MRSA, RPA

## Abstract

The increasing issue of infections caused by methicillin‐resistant *Staphylococcus aureus* (MRSA) necessitates rapid and reliable diagnostic methods. While existing RPA‐CRISPR/Cas12a platforms have demonstrated potential for MRSA detection, most rely on single‐gene targets or require multiple Cas enzymes. Here, we have developed a novel dual gene detection strategy that simultaneously detects the *S. aureus* specific *femA* gene and the methicillin‐resistant *mecA* gene in a single RPA‐CRISPR/Cas12a reaction. This integrated approach enables clear discrimination between MRSA and methicillin‐sensitive *Staphylococcus aureus* (MSSA) in just 30 min, with results visualized via both fluorescence and lateral flow strips. The assay exhibited high specificity (no cross‐reactivity with common pathogens) and a sensitivity of 10 copies/μL, comparable to qPCR. Validation with 39 clinical samples showed 100% concordance with antimicrobial susceptibility testing. Our dual‐gene RPA‐CRISPR/Cas12a platform represents a significant advancement in point‐of‐care MRSA diagnostics, offering enhanced accuracy and operational simplicity.

## 1 Introduction

In recent years, the emergence and spread of antibiotic resistance among bacteria have posed serious challenges to global public health and the medical field, leading to the emergence of numerous drug‐resistant strains [1, 2, 3]. Methicillin‐resistant *Staphylococcus aureus* (MRSA) is a gram‐positive bacterium and a drug‐resistant isolate of *S. aureus* [4, 5]. The prevalence of methicillin‐sensitive *Staphylococcus aureus* (MSSA) and MRSA has caused many medical and health problems [6, 7]. Infections of MSSA and MRSA can cause skin and soft tissue infections, as well as serious systemic illnesses, such as bacteremia, endocarditis, osteomyelitis, hemolytic pneumonia, and toxic shock syndrome, posing a great threat to the health and quality of life of patients [8, 9, 10, 11]. Under such circumstances, rapid and accurate detection of MSSA and MRSA becomes extremely important. Timely testing can help medical institutions take effective infection control measures, reduce the spread and cross infection of infections [12]. In addition, accurate detection results can also guide doctors in selecting the most suitable medication during treatment, improve efficacy, and reduce the risk of treatment failure and drug resistance [13].

New technologies for MSSA and MRSA detection are being actively researched and developed [9, 14, 15]. Two main methods are widely used, including the Kirby–Bauer method, which involves using cefoxitin paper sheets for screening tests and typically requires over 12 h for bacterial culture and identification, thus consuming a considerable amount of time [16, 17, 18]. The other is a molecular biology‐based nucleic acid assay, which takes less time than the standard method. Several DNA‐based molecular methods have been used for MRSA detection, including PCR, real‐time PCR, multiplex PCR, loop‐mediated isothermal amplification (LAMP), and recombinase polymerase amplification (RPA) [16, 19, 20, 21, 22, 23]. Besides, the emerging nanopore sequencing technology has been applied to identify a wide range of viral and bacterial pathogens from clinical samples due to its advantages of rapid library preparation and real‐time data acquisition [24, 25, 26]. While these methods have good sensitivity and specificity, each requires expensive equipment and a standardized laboratory and specialized technicians to operate, which is always difficult to achieve in areas where equipment is not widely available or in environments with very limited resources.

Lately, the combined utilization of RPA technology and CRISPR/Cas12a technology has provided new potential for the rapid detection of MRSA [27, 28]. This technology takes advantage of isothermal cyclic nucleic acid amplification, which can rapidly amplify target genes in a relatively short period of time, and combined with the highly specific cleavage enzyme activity of the CRISPR/Cas12a system, it enables the detection of nucleic acids by cleaving nearby non‐targeted reporter single‐stranded DNA [29]. The *femA* and *mecA* genes are two key genes in the genus *S. aureus* . The enzyme encoded by the *femA* gene plays an important role in bacterial cell wall synthesis and is a species‐specific gene for identifying *S. aureus* [30, 31, 32], while the *mecA* gene encodes the methicillin‐resistant associated protein penicillin binding protein 2a (PBP2a), which is a characteristic resistance gene of MRSA [33, 34]. By simultaneously detecting the *femA* and *mecA* genes, MSSA and MRSA can be accurately distinguished, which can better avoid false negative results caused by gene degradation, copy number differences, or amplification errors compared to single‐gene testing, thereby improving the reliability of the detection [35, 36].

At present, some studies have applied RPA and CRISPR/Cas12a for MRSA detection, and their strategies mainly include: single‐gene testing only for mecA [27]; some approaches require the use of multiple CRISPR systems, such as Cas12a combined with Cas13a, to achieve dual‐gene detection [37]; there have also been studies using other isothermal amplification techniques such as LAMP to bind with Cas12a [29]. These methods still face limitations in implementing dual gene detection, such as complex detection systems, dependence on multi enzyme systems, or relatively cumbersome operational steps. Therefore, this study aims to establish an RPA‐CRISPR/Cas12a dual gene detection platform based on a single Cas12a enzyme, which can simultaneously detect *femA* and *mecA* in the reaction, in order to achieve accurate and rapid identification of MRSA while simplifying the system. This method does not require complex instruments and is suitable for on‐site detection of different types of clinical samples.

## 2 Materials and Methods

### 2.1 Design of Primers, crRNAs, and ssDNA Reporter

All oligonucleotides, including RPA primers, crRNAs, and ssDNA reporter molecules, were synthesized by Sangon Biotech (Shanghai, China). Multiple pairs of RPA primers were designed using the target sequences of the *femA* and *mecA* genes at their three loci. The primer design followed with the principles stated in the TwistAmp Testing Design Manual (https://www.twistdx.co.uk/support/rpa‐assay‐design/). Additionally, the crRNA was designed using the CRISPRSCAN online tool, with the target sequence selected adjacent to the PAM site (https://www.crisprscan.org/sequence/). The 5′ end of ssDNA reporter molecule was modified with FAM, and the 3′ end was modified with BHQ1 (for fluorescence detection) or biotin (for lateral flow strip [LFS] detection), respectively. The sequences of RPA primers, crRNA, and ssDNA reporter molecules are listed in Table S1.

### 2.2 RPA Amplification

The reaction mixture was prepared according to the manufacturer's instructions (TwistDx, UK). Briefly, 12 μL of the provided buffer was used to resuspend the reaction pellet (which contains the polymerase and necessary ions). For a single‐gene RPA amplification, a primer mixture (0.5 μM each of *femA*‐F and *femA*‐R, or 0.5 μM each of *mecA*‐F and *mecA*‐R), 2 μL of extracted DNA, and 1 μL of MgOAc were added. The total reaction volume was adjusted to 50 μL with nuclease‐free water. A dual RPA assay, except for the adjusted primer concentration, was the same as a single RPA assay. The primer mixture consisted of *femA*‐F, *femA*‐R, *mecA*‐F, and *mecA*‐R, each at a final concentration of 0.4 μM.

### 2.3 CRISPR/Cas12a Detection

The CRISPR/Cas12a detection was performed in a 20 μL reaction system. The optimized final reaction contained: 100 nM LbaCas12a (NEB, USA), 100 nM target‐specific crRNA (Sangon Biotech, China), 500 nM ssDNA reporter (FAM/BHQ1‐labeled for fluorescence or FAM/Biotin‐labeled for LFS), 2 μL of 10× NEBuffer 2.1 (NEB, USA), 10 μL of RPA amplification product, and RNase‐free water up to 20 μL. For fluorescence detection, the mixture was incubated at 37°C in a real‐time PCR system (Applied Biosystems 7500) with fluorescence measurements taken at 3‐min intervals for 15 min. For LFS detection, the reaction was carried out at 37°C for 15 min, followed by applying the product to the strip (Milenia Hybridetect 1). Results were read after 3 min. The appearance of bands at both the control (C) and test (T) lines, or a single band at the T line, was interpreted as a positive result. A single band at the C line indicated a negative result.

### 2.4 Evaluation of Specificity

In this study, the standard strains MRSA (GDMCC1.771), *Acinetobacter baumannii* (GDMCC1.609), *Klebsiella pneumoniae* (GDMCC1.448) were provided by the Guangdong Microbial Culture Collection Center (GDMCC); *S. aureus* (BNCC186335), *Escherichia coli* (BNCC133264), *Pseudomonas* *aeruginosa* (BNCC337940) were provided by BeNa Culture Collection (BNCC). In addition, the detection of MRSA and MSSA in a mixture of multiple bacteria was also tested. Four combinations were set up: Combination 1 (MRSA + MSSA + *E. coli* + *P. aeruginosa* + *A. baumannii* + *K. pneumoniae* ), Combination 2 (MRSA + *E. coli* + *P. aeruginosa* + *A. baumannii* + *K. pneumoniae* ), Combination 3 (MSSA + *E. coli* + *P. aeruginosa* + *A. baumannii* + *K. pneumoniae* ), and Combination 4 ( *E. coli* + *P. aeruginosa* + *A. baumannii* + *K. pneumoniae* ).

Genomic DNA was extracted with the Bacterial Genomic DNA Extraction Kit (TIANGEN, Beijing, China) according to the kit instructions. Then RPA amplification and CRISPR/Cas12a detection were performed using optimized methods and conditions for RPA and CRISPR/Cas12a.

### 2.5 Evaluation of Sensitivity

Using the extracted genomic DNA of MRSA and MSSA as templates, the *femA* and *mecA* genes were amplified through RPA reaction. The RPA amplification products were then subjected to gel electrophoresis in a 3% agarose gel, followed by gel band excision and subsequent DNA purification using a gel extraction kit (TIANGEN, Beijing). The target fragments were ligated with the pESI‐blunt vector (Yeasen, Shanghai) to construct plasmid standards. The concentration of the constructed plasmid standards was converted to copies/μL and diluted in a 10‐fold gradient with a total of six concentrations (10^0^, 10^1^, 10^2^, 10^3^, 10^4^, 10^5^). Each of these six concentrations of DNA was used as a template for RPA amplification, followed by CRISPR‐fluorescence assay and CRISPR‐LFS assay.

### 2.6 Real‐Time PCR Detection

Commercialized *S. aureus mecA* gene dye‐based fluorescent quantitative real‐time PCR (qPCR) kit (Beijing Tianjingsha) was purchased for testing using Applied Biosystems 7500 real‐time PCR. Ten microliter of the reaction system was referenced to the kit instructions with slight modifications: 5 μL 2× SYBR qPCR Master Mix, 1 μL primer mix and 4 μL DNA or ultrapure water. The reaction program was 95°C for 5 min; 95°C for 10 s, 60°C for 40 s, 35 cycles. A standard curve for quantification was prepared by diluting the plasmid DNA of *mecA* in the kit into six concentration gradients (10^0^, 10^1^, 10^2^, 10^3^, 10^4^, 10^5^), which yielded a highly significant relationship (*R* ^2^ ≥ 0.99).

### 2.7 Clinical Samples

The study was approved by the Ethics Committee of Yuebei People's Hospital Affiliated to Shantou University Medical College (Code: YBEC‐KY(2023)‐(141)). Clinical samples were provided by the Department of Laboratory Medicine of Yuebei People's Hospital Affiliated to Shantou University Medical College, including 8 urine, 15 sputum, and 16 wound secretions. Sample pretreatment was performed. For urine samples, 1 mL of urine was centrifuged at 4°C, 6000 rpm for 10 min, the supernatant was discarded, the precipitate was retained, 1 mL of sterile saline was added to dissolve the precipitate, and the tube was tightly capped. For sputum samples, pick up about 1 mL of concentrated sputum into a labeled 15 mL centrifuge tube, add 1 mL of sputum digest (leagene) for homogenization, vortex and shake for 20 s, let it stand at 37°C for 20 min, add sterile PBS (pH 6.8) to about 10 mL, centrifuge at 3000 rpm for 15 min, discard the supernatant, and then resuspend the precipitate by adding 1 mL of PBS. For secretion labeled samples, 1 mL of sterile saline was placed in an EP tube, a swab with secretion was inserted into the centrifuge tube and spun, the swab was broken to cover the mouth of the tube tightly, and the tube was shaken vigorously to facilitate the elution of bacteria. A portion of the treated samples were inoculated onto Columbia blood plates and cultured at 37°C for 18–48 h. Bacterial identification and antimicrobial susceptibility testing were performed using the VITEK 2 system (bioMérieux, France), while genomic DNA was extracted from another portion of the samples using a microbial nucleic acid extraction kit (BIOG, BAIDAI) for subsequent RPA‐CRISPR/Cas12a, nanopore sequencing, and qPCR analyses.

### 2.8 16S rRNA Nanopore Sequencing

The 16S Barcoding Kit (SQK‐16S024, Oxford Nanopore Technologies) was used to construct the library. Ten ng of step 2.7 extracted genomic DNA was firstly quantified to 15 μL, and then 25 μL of long‐fragment high‐fidelity amplification enzyme mixture and 10 μL of labels and primers with special barcodes were added and mixed well. PCR amplification was performed. PCR amplification consisted of 95°C for 1 min, followed by 25 cycles of 95°C for 20 s, 52°C for 20 s, and 65°C for 2 min, ending at 65°C for 5 min. Amplicon concentration was determined using the Equalbit 1× dsDNA HS Assay Kit (Vazyme, Nanjing) and Qubit Flex (Invitrogen), and 10 cycles of amplification were performed for under‐concentration. Subsequently, the amplicons were purified by magnetic beads, the purified nucleic acids were quantified again, and the qualified libraries were combined together in a total volume of 10 μL, with a total sample size of 80 ng. Rapid adapter (1 μL) was added to the mixed libraries, and the libraries were left at 25°C for 20 min. The sequencing chip R9.4.1 was removed according to the instructions of the Ligation Sequencing Kit (SQK‐LSK110, Oxford Nanopore Technologies) to activate the chip, prepare the library and load it onto GridION (Oxford Nanopore Technologies) for nanopore sequencing.

### 2.9 Statistical Analysis

All data are expressed as mean ± standard deviation (*x* ± SD) of three parallel samples. The ImageJ analysis tool was used to examine the LFS band strength. Statistical analyses were performed by one‐way ANOVA using SPSS statistical software (version 22.0, IBM, USA). Statistical significance was set at 0.05.

## 3 Results

### 3.1 Principle of RPA‐CRISPR/cas12a Assay

**FIGURE 1:**
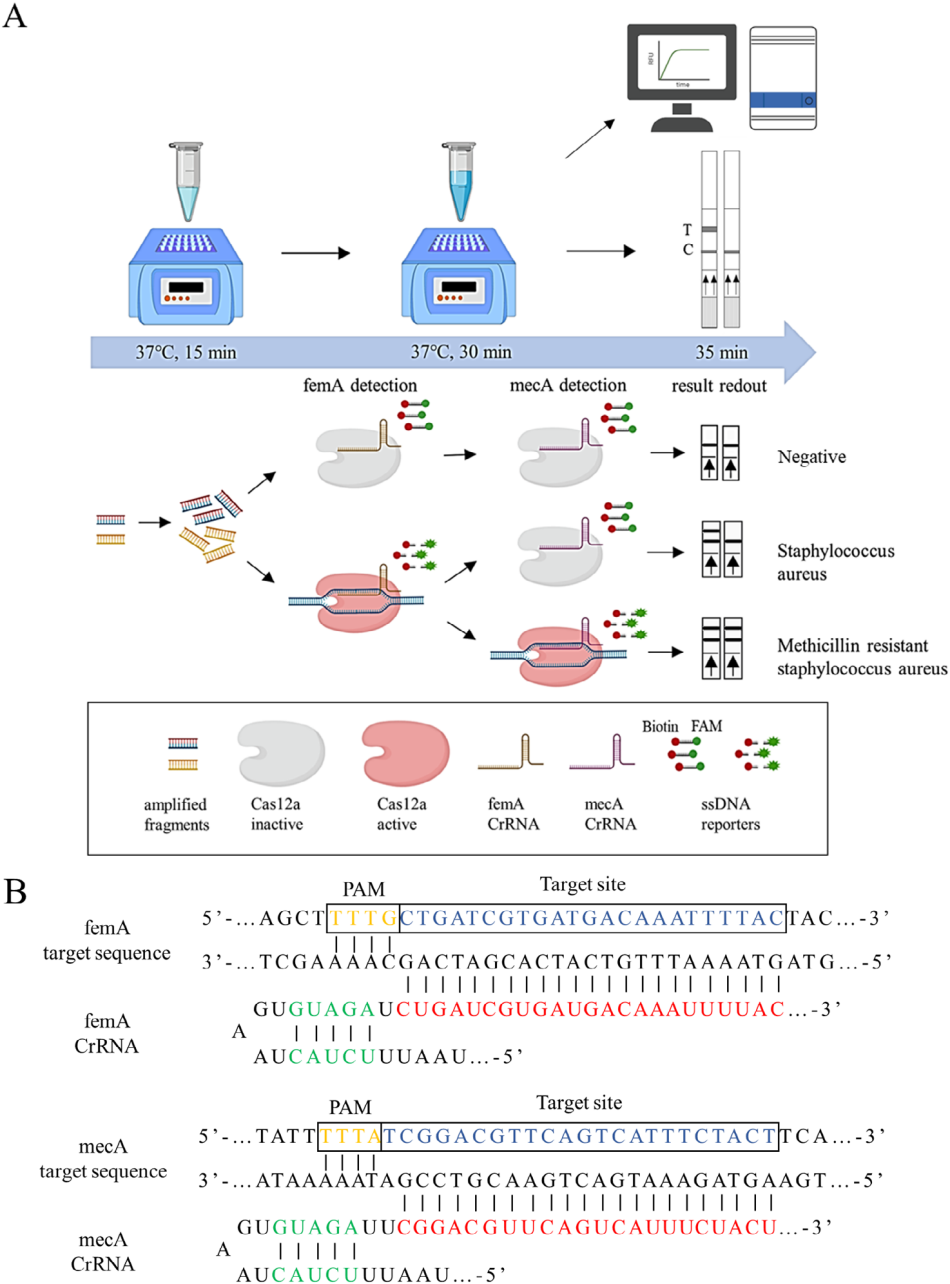
Schematic of RPA‐CRISPR/Cas12a detection. (A) Schematic diagram of the MRSA RPA‐CRISPR/Cas12a assay workflow. (B) Target sequences and crRNAs for CRISPR/Cas12a detection of *femA* and *mecA* genes.

The principle and flow of the RPA‐CRISPR/Cas12a assay are shown in Figure 1A. Firstly, *femA* and *mecA* genes were amplified simultaneously under the action of RPA primers and recombinase, and the target gene segment containing the PAM site (TTTV) was amplified exponentially under isothermal conditions at 37°C. The two genes were then detected separately, and the amplified products were added to an assay solution containing Cas12a, specific crRNA and ssDNA reporter gene (ssDNA labeled with fluorophores). Cas12a binds to a specific crRNA identifying dsDNA or ssDNA to form a Cas12a/crRNA/target DNA ternary complex, which triggers the trans‐cutting activity of Cas12a to nonspecifically cleave the ssDNA reporter gene, which can be detected either by fluorescent signal or LFS [38]. The whole detection process, including RPA amplification (15 min), CRISPR/Cas12a detection (15 min), and fluorescence detection or LFS color development (5 min), can be completed within 35 min. The target sequences and corresponding crRNAs for the femA and mecA genes are detailed in Figure 1B.

### 3.2 Construction of RPA Reaction

**FIGURE 2:**
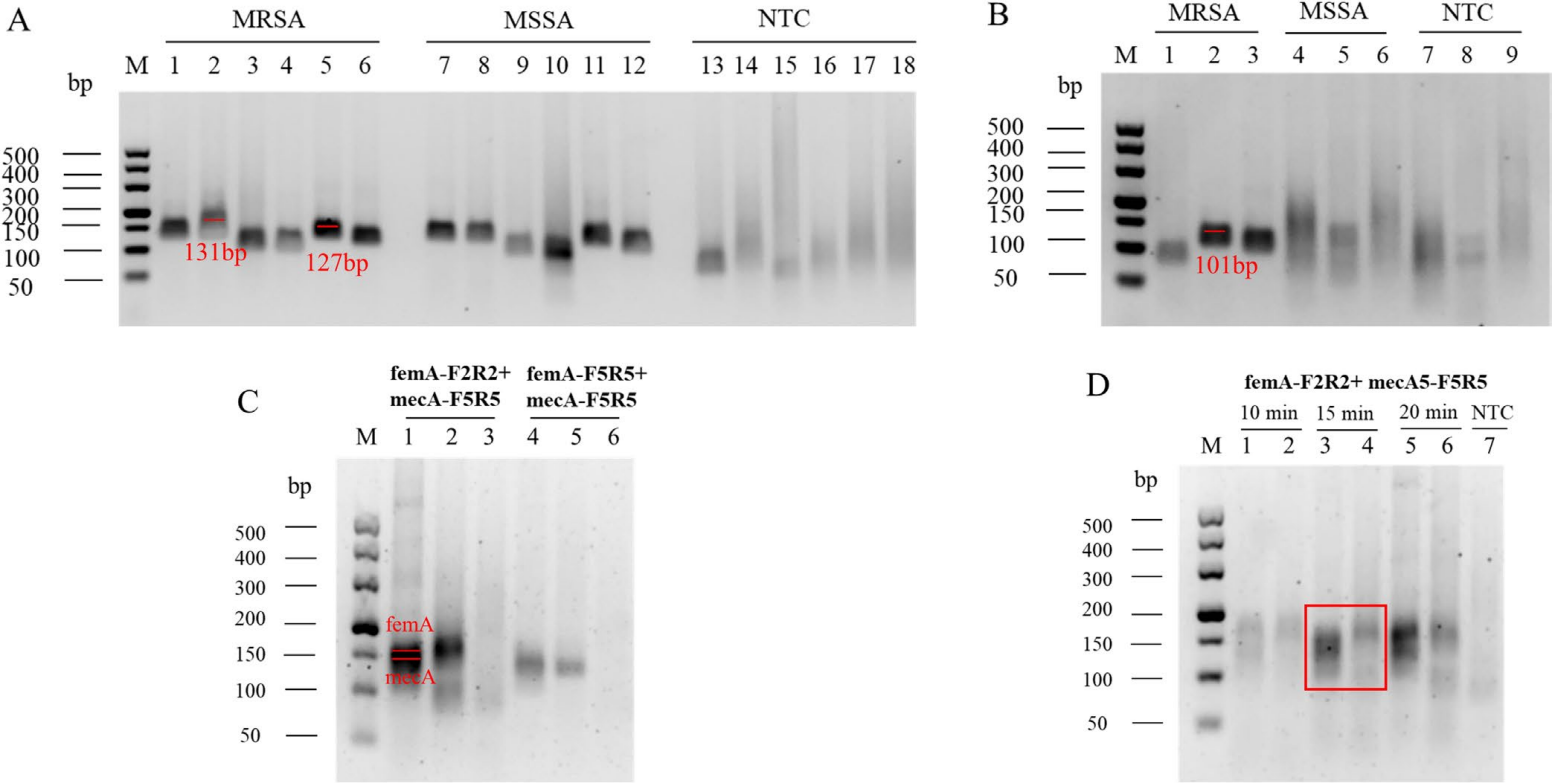
Selection of RPA primers and optimization of amplification conditions. (A) Selection of gene *femA* RPA primers. (B) Selection of gene *mecA* RPA primers. (C) RPA amplification of gene *femA* and *mecA*. The selected primers are indicated in red. (D) Optimization of RPA amplification conditions. The template for lane 135 is MRSA, and the template for lane 236 is MSSA.

Six pairs of primers for the *femA* gene and three pairs of primers for the *mecA* gene were initially designed for RPA amplification reactions (Table S1). In single‐gene RPA amplification, electropherogram results showed that primer F2R2 for *femA*, primer F5R5 for *femA*, and primer F5R5 for *mecA* amplified better (Figure 2A,B), so combinations of *femA*‐F2R2 + *mecA*‐F5R5 and *femA*‐F5R5 + *mecA*‐F5R5 were selected for dual‐gene amplification. In dual‐gene amplification, the combination *femA*‐F2R2 + *mecA*‐F5R5 amplified better than *femA*‐F5R5 + *mecA*‐F5R5 (Figure 2C), so *femA*‐F2R2 + *mecA*‐F5R5 was selected for optimization of RPA reaction conditions. The RPA reaction times of 10, 15, and 20 min were tested, and the results showed that there was already a more obvious target fragment at 15 min (Figure 2D). Uncropped gel images corresponding to the primer selection and optimization results in Figure 2A–D are provided in Figure S1A–C, respectively. In order to improve the assay efficiency and minimize the time required, the RPA reaction time was shortened to 15 min, and the optimized RPA reaction conditions were 37°C for 15 min.

### 3.3 Construction and Optimization of CRISPR Cas12a Detection System

**FIGURE 3:**
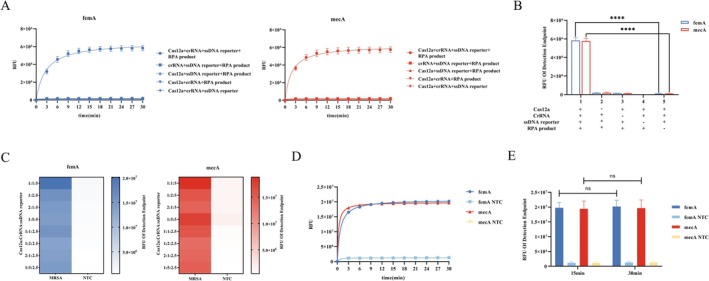
Combination of Cas12a and RPA for dual gene fluorescence detection and optimization of reaction conditions. Continuous fluorescence detection of *femA* and *mecA* genes. Test every 3 min for a total of 30 min. (A) Validation of trans‐cleavage of Cas12a by combinations of different components. (B) Endpoint fluorescence detection of *femA* and *mecA* genes in the combination of different components. (C) CRISPR‐fluorescence detection of *femA* and *mecA* genes in different ratios. The optimal ratio of Cas12a:CrRNA:SsDNA reporter = 1:1:5. (D) Continuous fluorescence detection of *femA* and *mecA* genes at the optimal ratio. (E) Endpoint fluorescence detection of *femA* and *mecA* genes at optimal ratios for 15 min and 30 min. Data are representative of at least three independent experiments (NTC: negative control, **** < 0.0001, *** < 0.001, ** < 0.01, * < 0.05, ns: not significant).

The functional components in the reaction mixture were confirmed by detecting the fluorescence intensity of each combination. As shown in Figure 3A,B, only the combination containing all components (CRISPR/Cas12a, crRNA, ssDNA reporter, and RPA product) showed a positive result, and the intensity of this fluorescence was significantly higher than the other reactions. This suggests that RPA‐amplified sequences can be accurately recognized by the Cas12a/crRNA complex and form the Cas12a‐crRNA‐RPA product complex, which activates the trans‐cleaving ability of Cas12a to collaterally cleave nearby ssDNA reporter genes, which plays a crucial role in the enhancement of the signal.

By comparing fluorescence intensities across a matrix of component ratios (Cas12a: 100 or 200 nM; crRNA: 100, 200, or 500 nM; ssDNA reporter: 250 or 500 nM), we determined that a 1:1:5 M ratio yielded the highest trans‐cleavage activity (Figure 3C). Further kinetic analysis showed that a 15‐min incubation at 37°C was sufficient to achieve maximal signal, with no significant gain from extending the reaction to 30 min (Figure 3D,E). Therefore, the conditions described in the Methods section (100 nM Cas12a, 100 nM crRNA, 500 nM ssDNA reporter, 37°C for 15 min) were adopted for all subsequent assays.

### 3.4 Evaluation of the Specificity of the RPA‐CRISPR/Cas12a Assay System

**FIGURE 4:**
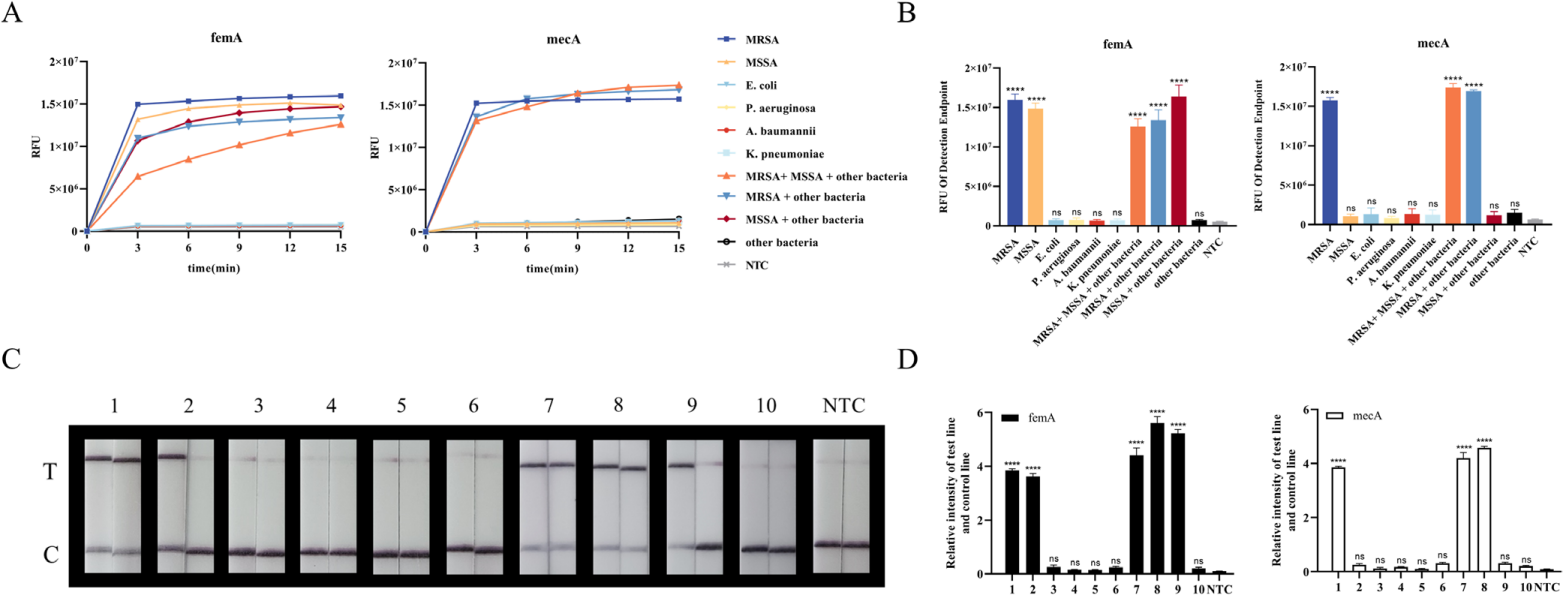
Determination of the specificity of RPA‐CRISPR/Cas12a detection of MRSA and MSSA using 4 common pathogenic bacteria. (A) Continuous fluorescence detection of *femA* and *mecA* genes in different pathogenic bacteria. Test every 3 min for a total of 15 min. (B) Endpoint fluorescence detection of *femA* and *mecA* genes in different pathogenic bacteria. (C, D) Visual detection of the *femA* and *mecA* gene of different pathogenic bacteria with lateral flow readout. The relative intensity of the test and control line was generated using ImageJ software. (1) MRSA, (2) MSSA, (3) *E. coli* , (4) *P. aeruginosa* , (5) *A. baumannii* , (6) *K. pneumoniae* , (7) MRSA + MSSA + *E. coli* + *P. aeruginosa* + *A. baumannii* + *K. pneumoniae* , (8) MRSA + *E. coli* + *P. aeruginosa* + *A. baumannii* + *K. pneumoniae* , (9) MSSA + *E. coli* + *P. aeruginosa* + *A. baumannii* + *K. pneumoniae* , (10) *E. coli* + *P. aeruginosa* + *A. baumannii* + *K. pneumoniae* . Data are representative of at least three independent experiments (NTC: negative control, **** < 0.0001, *** < 0.001, ** < 0.01, * < 0.05, ns: not significant).

To evaluate the specificity of the RPA‐CRISPR/Cas12a based detection system, comparative testing was conducted using the genomes of different bacteria, including *S. aureus* , *E. coli* , *P. aeruginosa* , *A. baumannii*, and *K. pneumoniae* . The results showed that the fluorescence‐based RPA‐CRISPR/Cas12a assay produced a strong fluorescent signal that could be observed during real‐time analysis. The results showed that the fluorescence intensity of MRSA, MSSA, and the combination *femA* gene containing MRSA or MSSA was significantly higher than that of other groups, and the fluorescence intensity of MRSA and the combination *mecA* gene containing MRSA was significantly higher than that of other groups (Figure 4A,B). In the RPA‐CRISPR/Cas12a assay based on LFS, clear bands on the test line of the *femA* gene were observed by naked eye only in MRSA, MSSA, and combinations containing MRSA or MSSA; clear bands on the test line of the *mecA* gene were observed in MRSA and combinations containing MRSA, which all produced significantly higher signal intensities than other groups (Figure 4C,D). A series of evaluations not only distinguished MRSA and MSSA well, but also showed no cross‐reactivity between pathogens, confirming the very good specificity of the RPA‐CRISPR/Cas12a‐based detection platform.

### 3.5 Evaluation of Sensitivity of RPA‐CRISPR/Cas12a Detection System

**FIGURE 5:**
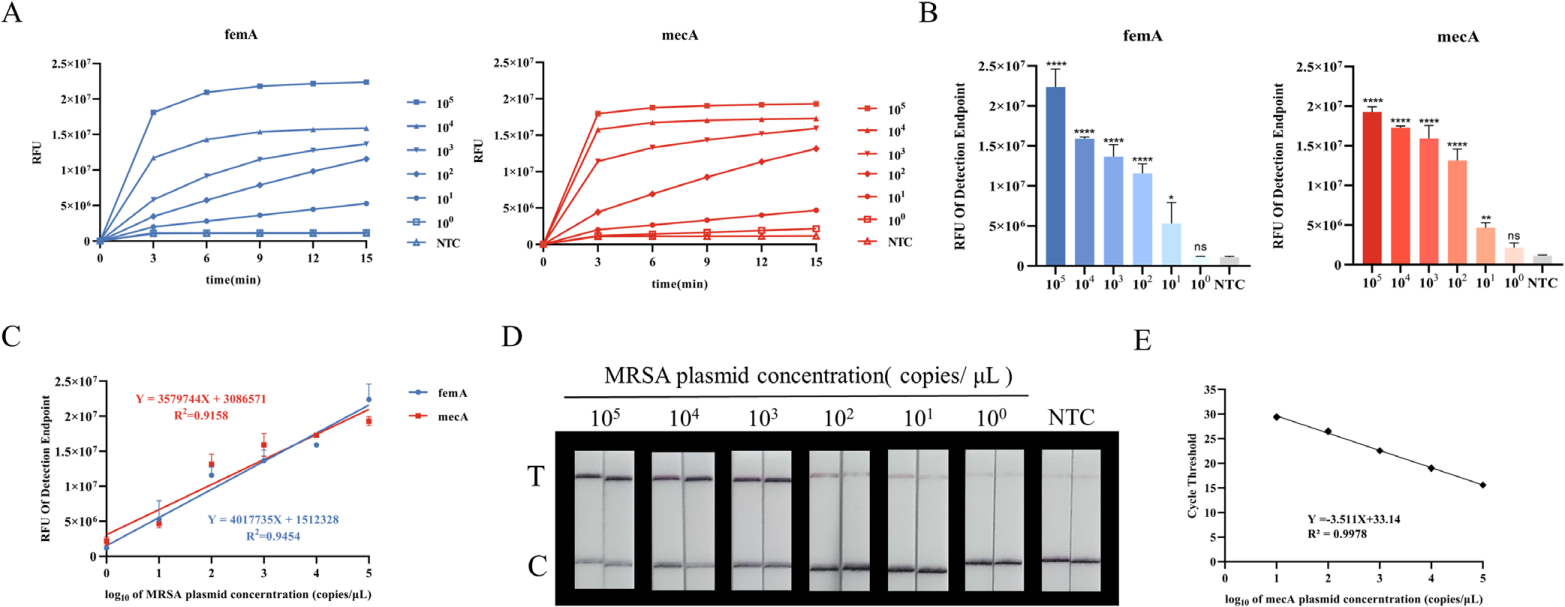
Determination of the sensitivity of RPA‐CRISPR/Cas12a detection. Different concentrations of *femA* and *mecA* plasmids. The gradient concentration of MRSA plasmids (10^0^ ∼ 10^5^ copies/μL) were used test. (A) Continuous fluorescence detection of *femA* and *mecA* genes in different gradient concentration. Test every 3 min for a total of 15 min. (B) Endpoint fluorescence detection of *femA* and *mecA* genes in different gradient concentration. (C) CRISPR‐fluorescence detection sensitivity analysis on *femA* and *mecA*, and the *R* ^2^ was 0.9454 and 0.9158. (D) Visual detection of the *femA* and *mecA* gene of MRSA plasmid dilution with lateral flow readout. (E) Quantitative Real‐time PCR amplification curves of *mecA* plasmids at different concentrations (10^1^ ∼ 10^5^ copies/μL), *R* ^2^ was 0.9978. Data are representative of at least three independent experiments (NTC: negative control, **** < 0.0001, *** < 0.001, ** < 0.01, * < 0.05, ns: not significant).

The sensitivity of the detection system was evaluated by detecting different concentrations of MRSA plasmids under optimal experimental conditions. The *femA* plasmid and *mecA* plasmid on MRSA were constructed. The concentrations of the constructed plasmid standards were converted to copies/μL and adjusted to 10^0^, 10^1^, 10^2^, 10^3^, 10^4^, 10^5^ copies/μL, which were used as detection templates to initiate the RPA‐CRISPR/Cas12a reaction. The assay results showed that when the concentration of the plasmid was between 10^1^ and 10^5^ copies/μL, it had obvious fluorescent signals and test line bands that could be observed by the naked eye, which were significantly different from the control group; however, the signal detected at a concentration of 1 copy/μ L cannot be distinguished from the control group (Figure 5A,B,D). Thus the method has a detection minimum of 10 copies/μL and it has a good linearity at intervals of 10^0^–10^5^ copies/μL (Figure 5C). In addition, the assay by fluorescent quantitative PCR was performed and the results were shown (Figure 5E). The stable detection of the *mecA* gene by fluorescence quantitative PCR method was at a minimum of 10 copies/μL. The results showed that the RPA‐CRISPR/Cas12a assay was comparable to fluorescent quantitative PCR in terms of detection sensitivity.

### 3.6 Clinical Sample Validation

Eight urine, 15 sputum, and 16 wound secretions were collected and processed briefly, and one portion of each sample was used for culture and antimicrobial susceptibility test, while the other portion of genomic DNA was extracted then used for RPA‐CRISPR/Cas12a, 16S rRNA nanopore sequencing, and qPCR.

**FIGURE 6:**
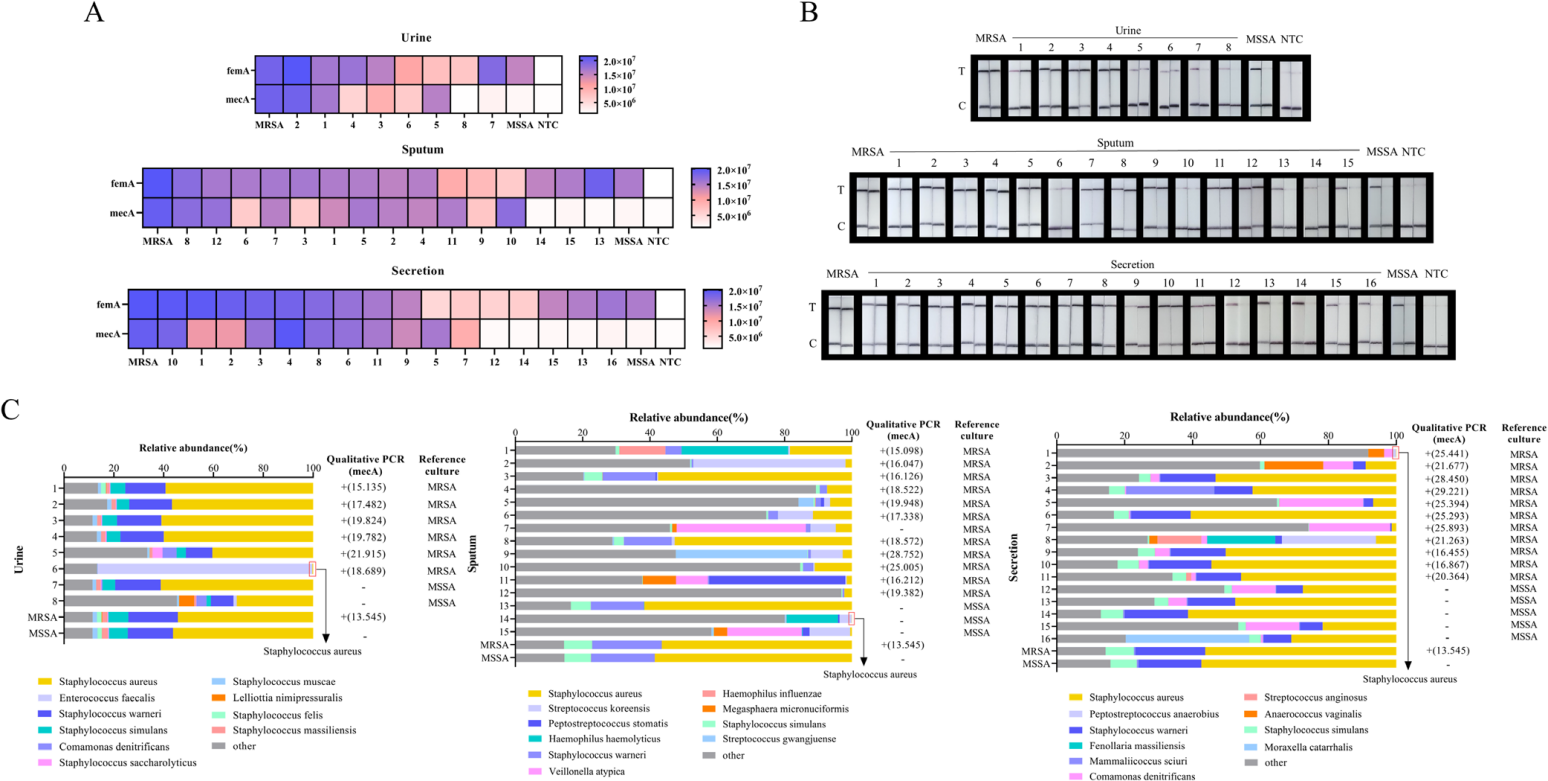
Application of RPA‐CRISPR/Cas12a assay to various clinical samples. (A) CRISPR‐fluorescence detection of *femA* and *mecA* genes in clinical samples. Blue indicates that the gene detected in the sample is positive and the shade of color is positively correlated with the fluorescence value. White color indicates that the gene detected in the sample is negative and the shade of white is negatively correlated with the fluorescence value. *femA* and *mecA* are both positive, indicating that the sample is MRSA. The *femA* is positive and *mecA* is negative, indicating that the sample is MSSA. (B) CRISPR‐LFS detection of *femA* and *mecA* genes in clinical samples. (C) Comparison of nanopore sequencing and qPCR data to the reference culture. In the left 100% stacked column, the small amount of Staphylococcus aureus in the figure is boxed in red. Data are representative of at least three independent experiments (NTC: negative control).

Twenty‐nine clinical samples identified by bacterial culture and antimicrobial susceptibility test were classified as MRSA, and 10 secretions were phenotypically classified as MSSA. RPA‐CRISPR/Cas12a detected the genomic DNA of the clinical samples, and the results of the assay were shown by the fluorescence signal intensity and the lateral flow test line. All 39 clinical samples were identified as *S. aureus* , of which 29 were MRSA and 10 were MSSA. The results showed that when using the RPA‐CRISPR/Cas12a method to detect clinical samples, for clinical samples isolated and identified as MRSA in antimicrobial susceptibility test, the fluorescence signals of *femA* and *mecA*, as well as the test strip test line signals, were significant and all results were positive. For clinical samples isolated and identified as MSSA in antimicrobial susceptibility test, the RPA‐CRISPR/Cas12a method detected significant fluorescence signals from *femA* and test strip signals, while the fluorescence signals from *mecA* and test strip signals were not significantly different from the control group. The *femA* result was positive, while the *mecA* result was negative (Figure 6A,B). The above test results show that this dual readout strategy is consistent and mutually validated.

In addition, these 39 clinical samples were also tested by nanopore sequencing. Since the resistance gene could not be detected by 16S rRNA nanopore sequencing, the resistance gene *mecA* was detected in the samples by qPCR, and the results of the culture were used as a reference (Figure 6C). The results showed that *S. aureus* was detected by nanopore sequencing in one of the sputum samples, while the drug resistance gene *mecA* could not be detected by qPCR, which was inconsistent with the reference culture MRSA. The detection results of all other types of samples are consistent with the reference culture. In addition to *S. aureus* , some other common clinical pathogens were detected by nanopore sequencing, such as *Enterococcus faecalis* , *Haemophilus haemolyticus* , *Parvimonas micra* , and *Peptostreptococcus anaerobius* . Detailed 16S rRNA nanopore sequencing analyses are presented in Tables S3–S5, and Ct averages for qPCR are presented in Table S2.

The RPA‐CRISPR/Cas12a assay was compared with the antimicrobial susceptibility test, nanopore sequencing, and qPCR methods (Table 1). Based on the identification results of the antimicrobial susceptibility test, the RPA‐CRISPR/Cas12a assay was positive for 29 MRSA clinical samples at a rate of 100.0% (29/29) and for 10 MSSA clinical samples at a rate of 100.0% (10/10), whereas nanopore sequencing was positive for MRSA at a rate of 96.6% (28/29) and for MSSA at a rate of 100.0% (10/10). The results demonstrated that our novel method is not only feasible in many types of clinical samples, but also comparable to nanopore sequencing and qPCR, and has the advantages of being less time‐consuming and low cost.

**TABLE 1:** Comparison of RPA‐CRISPR/Cas12a with nanopore sequencing, qPCR, culture, and antimicrobial susceptibility test for clinical validation.

	Culture and antimicrobial susceptibility test	CRISPR‐fluorescence	CRISPR‐lateral flow strip	Nanopore sequencing + qPCR
Sample coincidence rate	MRSA (29)	29 (100.0%)	29 (100.0%)	28 (96.6%)
	MSSA (10)	10 (100.0%)	10 (100.0%)	10 (100.0%)
Assay sample‐to‐result time	3–5 days	< 1 h	< 1 h	2 days
Sensitivity	Low	10 copies/μL	10 copies/μL	High
Specificity	High	High	High	Low
Complex laboratory infrastructure required	Yes	Yes	No	Yes
Cost	Inexpensive	Inexpensive	Inexpensive	Expensive

*Note:* Coincidence rate (CR) = 100.0% × [positive/total samples of the same type].

## 4 Discussion

At present, the methods for detecting MRSA in clinical practice, whether they are traditional cultivation methods, direct microscopy methods, antimicrobial susceptibility tests, or emerging PCR methods, all require professional equipment and skilled technical personnel, which limits the convenience and adaptability of testing. The RPA isothermal amplification technique used in this study is more popular compared to other nucleic acid amplification techniques due to its simplicity, sensitivity, extremely fast, low‐temperature constant temperature operation, simple instrument, and no need for multiple primers [39]. In this study, only a simple water bath capable of maintaining a constant temperature was used to complete the RPA pre amplification step within 15 min at 37°C. The reagent is freeze‐dried and stored in the reaction tube, making it easy to transport and use over long distances. The CRISPR/Cas biosensing system has been used in combination with nucleic acid testing in the field of in vitro diagnosis, and multiple diagnostic methods have been developed, such as SHERLOCK, HOLMES, and DETECTR [40, 41, 42]. The principle of CRISPR/Cas12a detection is that the Cas 12a/crRNA/target DNA forms a ternary complex, identifying the target sequence that is complementary to the crRNA sequence and adjacent to the appropriate prototype spacer motif (PAM, TTTN) [43]. After that, the trans‐cleavage activity of the CRISPR/Cas12a is activated, cutting the paired fluorescent groups and quencher labeled or fluorescent groups and biotin labeled ssDNA probes, and the results could be detected by fluorescence reading or LFS [44, 45]. Therefore, this study uses RPA amplification of target genes combined with CRISPR/Cas12a biosensing system to amplify signals for detection. Only inexpensive constant temperature equipment is needed to quickly detect MRSA, especially the RPA‐CRISPR/Cas12a LFS detection method, which has great potential for application in point‐of‐care testing (POCT). Moreover, after optimization, the detection process can be completed within 30 min, with RPA reaction lasting 15 min and CRISPR/Cas12a detection lasting 15 min, which is significantly faster and more advantageous than conventional detection methods.

It is noteworthy that MRSA is a drug‐resistant strain derived from *S. aureus* , and conventional detection methods struggle to differentiate between these two pathogens during testing unless antimicrobial susceptibility testing is performed after bacterial culture. The detection method developed in this study targets the species‐specific gene *femA* and the drug resistance gene *mecA*. A key advantage of this approach lies in its dual‐gene detection strategy, which not only enhances detection reliability but also effectively avoids false‐negative results caused by single‐gene deletion or amplification failure. This enables accurate discrimination between MRSA and MSSA, with no cross‐reactivity observed against other pathogens. Therefore, it holds significant value in clinical MRSA detection. In addition to its high specificity, the RPA‐CRISPR/Cas12a system demonstrates a limit of detection (LoD) of 10 copies/μL in terms of sensitivity. This is comparable to the sensitivity (10 copies/μL) reported for the previously described RAA‐Cas12a platform, while the detection time is reduced by half [46]. It was slightly lower than the limit of detection (LoD) of 10 aM (~6 copies/μL) for the nuc‐LAMP‐Cas12a platform and 1 aM (~1 copy/μL) for the *mecA*‐LAMP/Cas12a platform, but the assay time was shortened by 50 min [29]. Significant savings in assay time were achieved while achieving close sensitivity to that reported for similar assays. When compared with the sensitivity (5 copies/μL) of the one‐tube RPA‐CRISPR/Cas12a/Cas13a method for MRSA detection, although the sensitivity is slightly lower, only one type of Cas12a is needed to detect both the *femA* gene and the *mecA* gene at the same time, which reduces the reagents [37]. Detection requires only LFS, with no specialized equipment needed, greatly enhancing operational convenience and ease of use. The detection sensitivity is comparable to that of the Yanan's RPA‐cas12a platform (10–100 copies per reaction), while the detection time is slightly longer than that platform. However, a single reaction in our method enables effective differentiation between MRSA and SA, thereby improving the accuracy of the results [27].

In addition, the practicality of the assay system was evaluated using 39 different types of clinical samples, including urine, sputum, and secretions, which were also tested and compared with the methods of nanopore sequencing and qPCR. Nanopore sequencing has shown limitations in detecting drug resistance genes. In this study, although nanopore sequencing can detect a wider variety of pathogens than conventional methods, it is still necessary to detect the drug resistance gene *mecA* in the samples with the help of the qPCR method to make up for the shortcomings of nanopore sequencing. However, the assay results demonstrated 96.55% agreement with conventional methods. Specifically, in one sputum sample identified as MSSA by nanopore sequencing, qPCR failed to detect the *mecA* gene. This discrepancy may be attributed to local sequence variations within the *mecA* gene that impair primer binding, the presence of PCR inhibitors in the sample, or the possibility of a low‐copy heteroresistant bacterial population. Notably, the RPA‐CRISPR/Cas12a assay successfully detected both target genes, underscoring its robustness in complex clinical samples owing to high tolerance for sequence mismatches and strong resistance to amplification inhibitors. Furthermore, the RPA‐CRISPR/Cas12a platform offers operational simplicity, shorter turnaround time, and lower cost, making it well‐suited for high‐throughput rapid screening. These findings highlight the feasibility and superior suitability of our method for MRSA detection and analysis, supporting its potential for clinical application in pathogen identification and diagnosis.

Although the RPA‐CRISPR/Cas12a‐based assay for MRSA detection has demonstrated excellent performance under laboratory conditions, it is important to acknowledge several limitations of the current method. First, although we validated the assay with urine, sputum, and wound secretion samples, the cohort size and sample diversity remain limited. Future large‐scale, multicenter studies incorporating blood, nasal swabs, and other sterile site specimens are warranted to confirm broad applicability. Second, the current two‐step assay requires nucleic acid extraction prior to amplification. We are presently developing a streamlined, one‐pot protocol that integrates extraction‐free sample processing with RPA‐CRISPR/Cas12a detection to further simplify field deployment. Finally, while our dual‐gene approach improves detection accuracy, clinically relevant polymicrobial infections may require multiplexed detection of additional pathogens and resistance markers. Exploring rapid multiplex pathogen detection will also be a focus of our future work.

## 5 Conclusions

In conclusion, we developed a rapid and sensitive dual‐gene RPA‐CRISPR/Cas12a platform for MRSA detection. This method completes detection within 30 min using inexpensive instrumentation and provides results via both fluorescence and LFS readouts. The dual‐gene strategy targeting both *femA* and *mecA* not only ensures high specificity without cross‐reactivity but also enables clear discrimination between MRSA and MSSA. The assay demonstrates a sensitivity of 10 copies/μL, comparable to qPCR, and showed excellent concordance with antimicrobial susceptibility testing and nanopore sequencing in clinical validation. Although further optimization and larger‐scale studies are warranted, this integrated platform holds strong potential for point‐of‐care and clinical diagnostic applications.

## Author Contributions

Involvement in conception and design of the study: Pingsen Zhao and Lei Chen. Acquisition of the data: Lei Chen, Pingsen Zhao, Jun Luo, and Helin Zhang. Analysis and interpretation of the data: Lei Chen, Pingsen Zhao, Jun Luo, and Helin Zhang. Substantial involvement in the writing and/or revision of the article: Pingsen Zhao and Lei Chen. Responsible for content of the manuscript including data and analysis: Pingsen Zhao. All authors read and approved the final manuscript.

## Funding

This study was supported by Natural Science Foundation of Guangdong Province, China (Grant No.: 2025A1515010579 to Prof. Pingsen Zhao), Shaoguan Municipal Science and Technology Program, China (Grant Nos.: 211102114530659 and 220610154531525 to Prof. Pingsen Zhao); Shaoguan Engineering Research Center for Research and Development of Molecular and Cellular Technology in Rapid Diagnosis of Infectious Diseases and Cancer Program, China (Grant No.: 220525096180441 to Prof. Pingsen Zhao); Research Fund for Joint Laboratory for Digital and Precise Detection of Clinical Pathogens, Yuebei People's Hospital Affiliated to Shantou University Medical College, China (Grant No.: KEYANSHEN (2023) 01 to Prof. Pingsen Zhao); Research Project for Outstanding Scholar of Yuebei People's Hospital Affiliated to Shantou University Medical College, China (Grant No.: RS202001 to Prof. Pingsen Zhao); Research Project for Guangdong Provincial Clinical Research Center for Laboratory Medicine, China (Grant No.: 2023B110008 to Prof. Pingsen Zhao).

## Ethics Statement

This study has been approved by the Ethics Committee of Yuebei People's Hospital Affiliated to Shantou University Medical College (Code: YBEC‐KY(2023)‐(141)), and all patients or their legal representatives have signed informed consent. Additionally, all patients or their legal representatives have signed the informed consent form.

## Consent

The authors have nothing to report.

## Conflicts of Interest

The authors declare no conflicts of interest.

## Supporting information


**Figure S1:** Uncropped gel images of selected RPA primers and optimized amplification conditions. (A) Corresponding to uncropped glue figure in Figure 2A. (B) corresponding to uncropped glue figure in Figure 2B. (C) corresponding to uncropped glue figure in Figure 2C,D.

**Table S1:** Sequences used in this experiment.

**Table S2:** Clinical validation of qPCR detection for the MRSA.

**Table S3:** 16S rRNA nanopore sequencing in urine.
**Table S4:** 16S rRNA nanopore sequencing in sputum.
**Table S5:** 16S rRNA nanopore sequencing in secretion.

## Data Availability

The data that support the findings of this study are available from the corresponding author upon reasonable request.
