# Supplementary material for: RPA Combined With CRISPR/Cas12a for Rapid and Ultrasensitive Detection Dual‐Gene of Methicillin‐Resistant Staphylococcus aureus (MRSA)
Source: J Mol Recognit. 2026 Apr 19;39:e70035. doi: 10.1002/jmr.70035 (PMC13092367; doi:10.1002/jmr.70035)
Supplement: Supplementary file 1 — Figure S1: Uncropped gel images of selected RPA primers and optimized amplification conditions. (A) Corresponding to uncropped glue figure in Figure 2A. (B) corresponding to uncropped glue figure in Figure 2B. (C) corresponding to uncropped glue figure in Figure 2C,D. [file JMR-39-e70035-s001.docx]

| 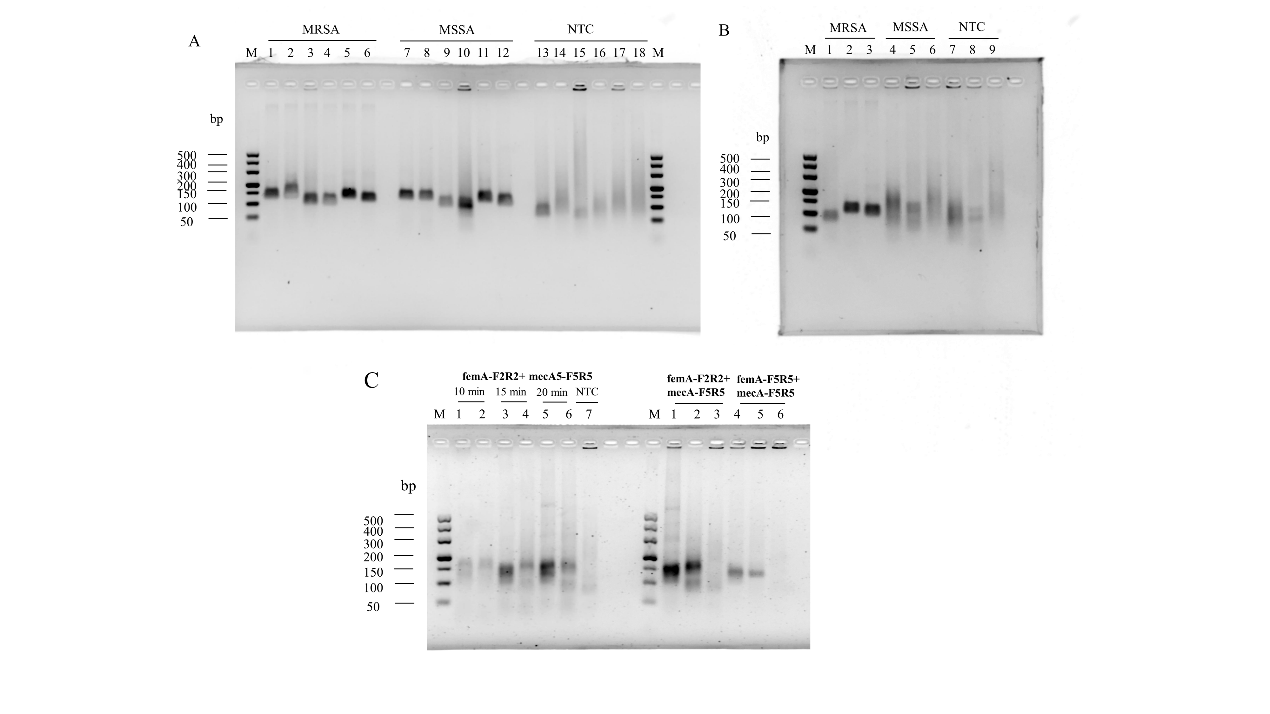 |
| --- |
| Figure S1. Uncropped gel images of selected RPA primers and optimised amplification conditions.  (A) corresponding to uncropped glue figure in Figure 2A. (B) corresponding to uncropped glue figure in Figure 2B. (C) corresponding to uncropped glue figure in Figure 2C and 2D. |
