# Supplementary material for: RPA Combined With CRISPR/Cas12a for Rapid and Ultrasensitive Detection Dual‐Gene of Methicillin‐Resistant Staphylococcus aureus (MRSA)
Source: J Mol Recognit. 2026 Apr 19;39:e70035. doi: 10.1002/jmr.70035 (PMC13092367; doi:10.1002/jmr.70035)
Supplement: Supplementary file 2 — Table S1: Sequences used in this experiment. [file JMR-39-e70035-s002.docx]

Table S1. Sequences used in this experiment

| Primer Name | Sequence 5’-3’ | Amplification length |
| --- | --- | --- |
| femA-F1 | GATGACATCATTAAAAATATGGATGGACTTAG | 131 bp |
| femA-R1 | GACGTATCTTCCATAAATGATCTAAAAATTGG |  |
| femA-F2 | TTTAGATCATTTATGGAAGATACGTCAGAATC | 131 bp |
| femA-R2 | TCATCAAAGTTGATATACGCTAAAGGTACTAA |  |
| femA-F3 | CCAATTTTTAGATCATTTATGGAAGATACGTC | 103 bp |
| femA-R3 | AACGGTCTTTGTAATATTTTAAGCGATTGTAG |  |
| femA-F4 | ATCATTTATGGAAGATACGTCAGAATCAAAAG | 101 bp |
| femA-R4 | GTACTAACAAACGGTCTTTGTAATATTTTAAGC |  |
| femA-F5 | CATTAAATCATGGCATTGACCGTTATAATTTC | 127 bp |
| femA-R5 | CCAACATATTCAATAATTTCAGCATTGTAACC |  |
| femA-F6 | CCGTTATAATTTCTATGGTGTTAGTGGTAAAT | 115 bp |
| femA-R6 | AAAGTCACCAACATATTCAATAATTTCAGC |  |
| mecA-F2 | CTCTAGCAAAGAAAATGTTATCTGATGATTCT | 83 bp |
| mecA-R2 | CGTTACAAGATATGAAGTGGTAAATGGTAATA |  |
| mecA-F3 | AAAATGGATAATCACTTGGTATATCTTCACC | 116 bp |
| mecA-R3 | ATCATCAGATAACATTTTCTTTGCTAGAGTAG |  |
| mecA-F5 | GATCCTGAATGTTTATATCTTTAACGCCTAA | 101 bp |
| mecA-R5 | CAGTTATATTTCTAAAAGCGATAATGGTGAAG |  |
| femA-crRNA2 | UAAUUUCUACUAAGUGUAGAUCUGAUCGUGAUGACAAAUUUUAC |  |
| mecA-crRNA5 | UAAUUUCUACUAAGUGUAGAUUCGGACGUUCAGUCAUUUCUACU |  |
| FQ-ssDNA reporter | 5’6-FAM-TTATTATT-3’BHQ1 |  |
| FB-ssDNA reporter | 5’6-FAM-TTATTATT-3’Botin |  |
