# Supplementary material for: RPA Combined With CRISPR/Cas12a for Rapid and Ultrasensitive Detection Dual‐Gene of Methicillin‐Resistant Staphylococcus aureus (MRSA)
Source: J Mol Recognit. 2026 Apr 19;39:e70035. doi: 10.1002/jmr.70035 (PMC13092367; doi:10.1002/jmr.70035)
Supplement: Supplementary file 3 — Table S2: Clinical validation of qPCR detection for the MRSA. [file JMR-39-e70035-s004.docx]

Table S2. Clinical validation of qPCR detection for the MRSA

| MSSAmple type | Number | Ct mean | Qualitative | Culture |
| --- | --- | --- | --- | --- |
| Urine | 1 | 15.135 | + | MRSA |
|  | 2 | 17.482 | + | MRSA |
|  | 3 | 19.824 | + | MRSA |
|  | 4 | 19.782 | + | MRSA |
|  | 5 | 21.915 | + | MRSA |
|  | 6 | 18.689 | + | MRSA |
|  | 7 | / | - | MSSA |
|  | 8 | / | - | MSSA |
| Sputum | 9 | 15.098 | + | MRSA |
|  | 10 | 16.047 | + | MRSA |
|  | 11 | 16.126 | + | MRSA |
|  | 12 | 18.552 | + | MRSA |
|  | 13 | 19.984 | + | MRSA |
|  | 14 | 17.338 | + | MRSA |
|  | 15 | / | - | MRSA |
|  | 16 | 18.572 | + | MRSA |
|  | 17 | 28.752 | + | MRSA |
|  | 18 | 25.005 | + | MRSA |
|  | 19 | 16.212 | + | MRSA |
|  | 20 | 19.382 | + | MRSA |
|  | 21 | / | - | MSSA |
|  | 22 | / | - | MSSA |
|  | 23 | / | - | MSSA |
| Secretion | 24 | 25.441 | + | MRSA |
|  | 25 | 21.677 | + | MRSA |
|  | 26 | 28.450 | + | MRSA |
|  | 27 | 29.221 | + | MRSA |
|  | 28 | 25.394 | + | MRSA |
|  | 29 | 25.293 | + | MRSA |
|  | 30 | 25.893 | + | MRSA |
|  | 31 | 21.263 | + | MRSA |
|  | 32 | 16.455 | + | MRSA |
|  | 33 | 16.867 | + | MRSA |
|  | 34 | 20.364 | + | MRSA |
|  | 35 | / | - | MSSA |
|  | 36 | / | - | MSSA |
|  | 37 | / | - | MSSA |
|  | 38 | / | - | MSSA |
|  | 39 | / | - | MSSA |
| MRSA | 40 | 13.545 | + |  |
| MSSA | 41 | / | - |  |
| NTC | 42 | / | - |  |
